# Practice disparities in palliative radiation therapy for bone metastases: insights from the Shizuoka Kokuho database study

**DOI:** 10.1007/s11604-025-01938-8

**Published:** 2026-01-14

**Authors:** Yuhei Miyasaka, Yoko Sato, Hideyuki Harada, Katsumasa Nakamura, Tatsuya Ohno, Seiichiro Yamamoto

**Affiliations:** 1 Graduate School of Public Health, Shizuoka Graduate University of Public Health, 4-27-2 Kitaando, Aoi-ku, Shizuoka, Shizuoka 420-0881 Japan; 2 Gunma University Heavy Ion Medical Center, 3-39-22, Showa-Machi, Maebashi, Gunma 371-8511 Japan; 3 Department of Radiation Oncology, Gunma University Graduate School of Medicine, 3-39-22, Showa-Machi, Maebashi, Gunma 371-8511 Japan; 4 Division of Radiation Therapy, Shizuoka Cancer Center, 1007 Shimonagakubo, Nagaizumi-cho, Sunto-gun, Shizuoka 411-8777 Japan; 5 Department of Radiation Oncology, Hamamatsu University School of Medicine, 1-20-1 Handayama, Chuo-ku, Hamamatsu, Shizuoka 431-3192 Japan

**Keywords:** Bone neoplasms, Healthcare disparities, Neoplasm metastasis, Palliative care, Radiotherapy

## Abstract

**Purpose:**

Palliative radiation therapy (pRT) is a standard treatment option for bone metastases from malignancies and is recommended in current guidelines. Patients should have an equal opportunity to receive pRT regardless of their residential address or the location of the treatment facility; however, the frequency of use of pRT varied across regions and healthcare systems in studies in North America. This study aimed to examine the disparity in Japan.

**Materials and methods:**

We conducted a historical cohort study using a regional population-based cohort that included medical insurance data to examine practice disparities in pRT for patients diagnosed with bone metastases who received bone-modifying agents (BMA). The analyses focused on the secondary medical areas (SMA) where the patients lived and facilities where they were treated.

**Results:**

Overall, 6289 patients were included in our study, and 39.6% received pRT. The regional disparity in the proportion of pRT delivery was not large, despite some areas having no facilities with RT devices. However, differences among facilities where BMA was initiated were substantial. The BMA facilities with RT devices had a higher proportion of pRT than those without RT devices. In addition, the difference was wide among the facilities with RT devices. Patients treated with BMA in designated cancer hospitals were likely to receive pRT than those of other hospitals.

**Conclusion:**

This population-based study using medical claims data demonstrated practice disparities in pRT for bone metastases, particularly at the facility level. To improve this disparity, facility-level strategies seem to be more effective than SMA-level approaches.

**Supplementary Information:**

The online version contains supplementary material available at 10.1007/s11604-025-01938-8.

## Introduction

Bone metastases are common in patients with advanced malignancies, especially those with primary cancers of the lung, prostate, and breast, contributing to a substantial healthcare burden. An epidemiological analysis using data from the Surveillance, Epidemiology, and End Results program estimated an annual incidence of 18.8 cases of bone metastases per 100,000 persons [1].

The management of bone metastases focuses on palliating symptoms, preventing skeletal-related events (SREs) and improving the quality of life. Among various treatment modalities, palliative radiation therapy (pRT) is a standard intervention for the relief of pain associated with bone metastases. The effectiveness of pRT in improving patients’ quality of life is well-documented, with pain relief observed in approximately 60–80% of patients [2]. Furthermore, a randomized controlled trial demonstrated the potential benefits of prophylactic radiation therapy (RT) for asymptomatic bone metastases with a high risk of bone fractures, showing a reduction in the incidence of SREs and potential improvements in survival outcomes [3].

Considering the benefits of pRT, all patients should have an equal opportunity to receive pRT appropriately, regardless of the area in which they live or facility where they are treated. However, the use of pRT in clinical practice varies widely across different regions and healthcare systems. Previous population-based studies in North America have highlighted significant disparities in access to and delivery of pRT [4–7]. Huang et al. reported that socioeconomic status, diagnostic facilities, and residential areas were associated with pRT delivery in the Ontario Cancer Registry between 1986 and 1995 [4]. Furthermore, Mackillop and Kong demonstrated that on-site RT availability in diagnostic facilities was associated with the proportion of pRT in the same registry from 2006 to 2010 [7]. In regions other than North America, comparable data on pRT utilization are scarce, leaving a critical knowledge gap regarding the practice differences in pRT.

In Japan, the Ministry of Health, Labour, and Welfare (MHLW) has established the Basic Plan to Promote Cancer Control Programs and set the equalization of cancer treatment as one of its goals. Therefore, the MHLW and Shizuoka Prefecture have developed designated cancer care hospitals [8]. However, the actual status of pRT equalization in bone metastases remains unknown.

This study addressed this disparity by examining the proportion of pRT deliveries in patients with bone metastases according to their residential areas or facilities where they were treated, using a Japanese regional population-based cohort. These findings may be helpful when considering policies and interventions aimed at equalizing pRT delivery for patients with bone metastases.

## Material and methods

### Study design and data source

We performed a historical population-based cohort study using the Shizuoka Kokuho Database (SKDB), version 2024-2 [9, 10]. The SKDB includes monthly administrative claims data for residents of Shizuoka Prefecture between April 2012 and September 2022. Shizuoka Prefecture is in central Japan and includes both rural and urban areas. The total number of patients treated with RT per institution in Shizuoka Prefecture was at the average level in Japan [11]. We accessed the SKDB for research purposes between November 3 and 5, 2024.

### Study population and observation

**Fig. 1:**
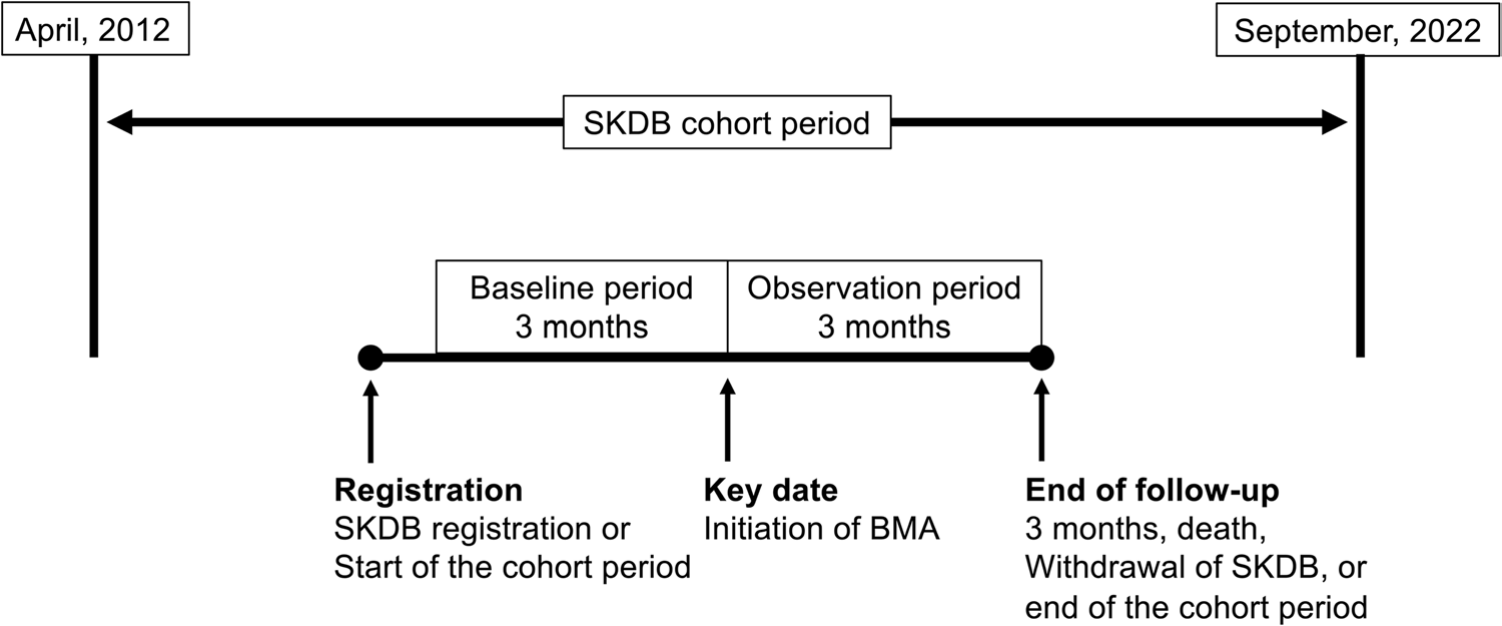
Study schema. SKDB, Shizuoka Kokuho Database; BMA, bone-modifying agents

The present study included patients with bone metastases, defined by the International Classification of Disease, tenth edition (ICD-10) code C79.5″ Secondary malignant neoplasm of bone and bone marrow”, treated with bone-modifying agents (BMA) specifically indicated for bone diseases from malignancies. The BMA included zoledronic acid and denosumab, however, BMA for osteoporosis was excluded (Table S1). Baseline characteristics were obtained within 3 months backward from the month of BMA initiation. The ICD-10 codes for malignancies obtained during the study period are summarized in Table S2. The design diagram of this study is shown in Fig. 1.

The key date was set as the date of initiation of BMA administration (new user design [12], a wash-out period of 3 months), and the follow-up period was limited to 3 months from the key date. RT during this period was defined as pRT. Intensity-modulated radiation therapy (IMRT), stereotactic body radiation therapy (SBRT), particle therapy, brachytherapy, and radioisotope therapy were excluded to ensure that the purpose of the RT was limited to palliation. The medical procedure codes for the RT are listed in Table S3. RT 3 months after the key date was also assessed to examine the validity of the pRT definition.

The proportion of patients who underwent pRT was evaluated across secondary medical areas (SMA) where the patients lived. The SMA is an administrative unit in Japan designed to provide general medical services. There are eight SMA in Shizuoka Prefecture: Seibu, Chutoen, Shida-Haibara, Shizuoka, Fuji, Sunto-Tagata, Atami-Ito, and Kamo. The proportion of pRT was evaluated based on the facilities where BMA was initiated. Subsequent analyses were conducted by categorizing these facilities according to the presence or absence of RT devices and whether they were designated cancer care hospitals.

### Statistical analysis

Tabular data were evaluated using the Fisher’s exact test. The Wilson score interval was used to demonstrate the 95% confidence interval (95% CI). Multivariate logistic regression analysis was used to adjust for variables. All analyses and visualizations were performed using R version 4.4.1 (R Core Team, Vienna, Austria) [13] or Microsoft Excel (Microsoft, WA, USA).

## Results

### Descriptions of characteristics and the pRT delivery in the entire cohort

Of the 2,267,253 individuals, 6,289 met our criteria for potential indications for pRT for bone metastasis (Table 1, Fig. S1). Among them, 3,140 (49.9% [95% CI: 48.7– 51.2%]) patients received RT after BMA initiation, and 2,490 (39.6% [95% CI 38.4–40.8%]) underwent pRT (Table S4, Fig. S2). The proportions of pRT according to patient factors are summarized in Table 2.

**Table 1:** Baseline characteristics of the entire cohort (n = 6289)

Factors	n (%)
*Gender*	
Men	3730 (59%)
Women	2559 (41%)
*Age at BMA initiation*	
< 60	439 (7.0%)
60 ≤ , < 75	3022 (48%)
75 ≤	2828 (45%)
*BMA*	
Denosumab	3322 (53%)
Zoledronic acid	2976 (47%)
*Malignant neoplasm*	
Head and neck	51 (0.8%)
Esophagus	96 (1.5%)
Stomach	124 (2.0%)
Colorectum	211 (3.4%)
Kidney	184 (2.9%)
Liver	196 (3.1%)
Pancreas	91 (1.4%)
Lung	1722 (27%)
Skin	22 (0.3%)
Breast	726 (12%)
Uterine cervix	31 (0.5%)
Endometrial	32 (0.5%)
Ovarian	15 (0.2%)
Prostate	819 (13%)
Bladder	94 (1.5%)
Thyroid	31 (0.5%)
Malignant lymphoma	42 (0.7%)
Multiple myeloma	104 (1.7%)
Others/ Unknown	1196 (19%)
*SMA*	
Seibu	1292 (20.5%)
Chutoen	798 (12.7%)
Shida-Haibara	694 (11.0%)
Shizuoka	1229 (19.5%)
Fuji	616 (9.8%)
Sunto-Tagata	1135 (18.0%)
Atami-Ito	340 (5.4%)
Kamo	185 (2.9%)
*RT availability at the facility for BMA*	
Yes	5587 (88.8%)
No	702 (11.2%)

BMA, bone-modifying agent; SMA, secondary medical area; RT, radiation therapy

**Table 2:** Numbers and proportions of the delivery of pRT

	Numbers of pRT N = 2490	Proportions of pRT (%)	95% CI
*Gender*			
Men	1482	39.7	38.1–41.3
Women	1008	39.4	37.5–41.3
*Age at BMA initiation*			
< 60	176	40.1	35.6–44.8
60 ≤ , < 75	1253	41.5	39.8–43.3
75 ≤	1061	37.5	35.7–39.3
*BMA*			
Denosumab	3322	36.8	35.2–38.5
Zoledronic acid	2976	42.6	40.8–44.4
*Malignant neoplasm*			
Head and neck	30	58.8	45.1–71.2
Esophagus	56	58.3	48.3–67.7
Stomach	55	44.4	36.0–53.2
Colorectum	114	54.0	47.3–60.6
Kidney	99	53.8	46.6–60.9
Liver	124	63.3	56.4–69.7
Pancreas	47	51.6	41.5–61.6
Lung	913	53.0	50.6–55.3
Breast	221	30.4	27.2–33.8
Uterine cervix	13	41.9	26.4–59.2
Endometrial	17	53.1	36.4–69.1
Prostate	157	19.2	16.6–22.0
Bladder	46	48.9	39.0–58.8
Thyroid	10	32.3	18.6–49.9
Malignant lymphoma	27	64.3	49.2–77.0
Multiple myeloma	42	41.2	31.5–50.0
Others/ Unknown	301	25.2	22.8–27.7

pRT, palliative radiation therapy; CI, confidence interval; BMA, bone-modifying agent

### Delivery of pRT by the SMA and facility where BMA was initiated

**Fig. 2:**
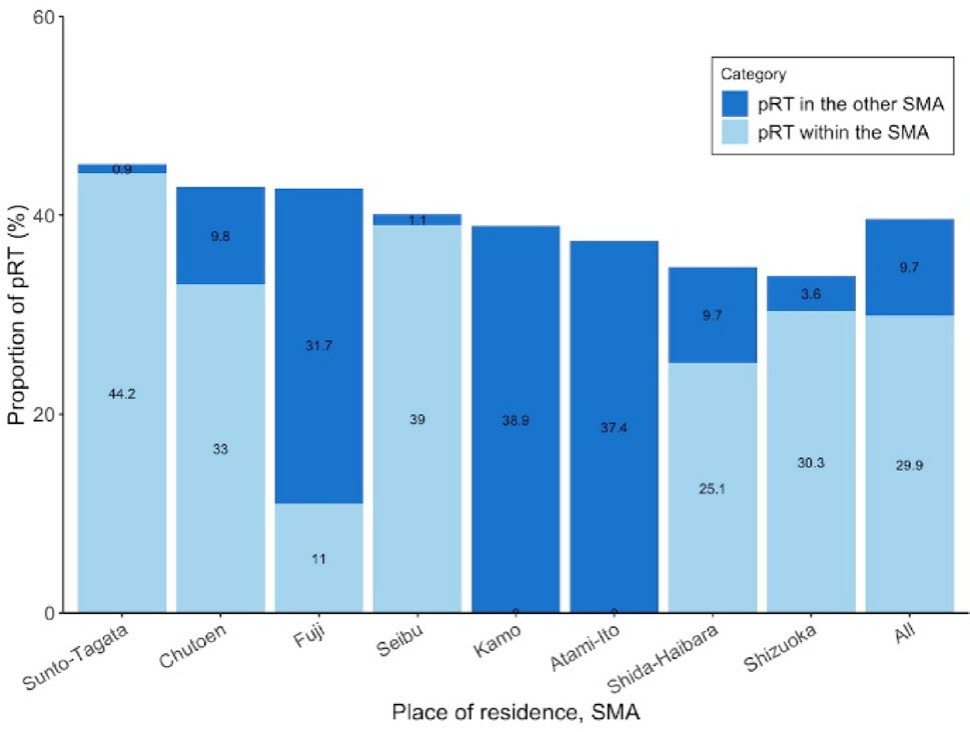
Proportion of patients who underwent pRT by SMA. Numbers in the plotted areas represent individual data. pRT, palliative radiation therapy; SMA, secondary medical area

The proportion of patients treated with pRT was investigated across SMA (Fig. 2). The proportion was 40.1% (95% CI 37.5–42.8%) in Seibu, 42.8% (95% CI 39.3–46.2%) in Chutoen, 34.8% (95% CI 31.3–38.3%) in Shida-Haibara, 33.9% (95% CI 31.2–36.5%) in Shizuoka, 42.7% (95% CI 38.9–46.6%) in Fuji, 45.1% (95% CI 42.2–48.0%) in Sunto-Tagata, 37.4% (95% CI 32.4–42.7%) in Atami-Ito, and 38.9% (95% CI 32.2–46.1%) in Kamo. As no RT facilities were in Atami-Ito and Kamo, all patients in these areas underwent pRT in other areas. Residents receiving pRT in areas other than their residential areas in Fuji, Shida-Haibara, Chutoen, and Shizuoka were 74.2% (95% CI 68.6–79.1%), 27.9% (95% CI 22.6–33.9%), 22.9% (95% CI 18.8–27.6%), 10.6% (95% CI 8.0–13.9%), respectively.

**Fig. 3:**
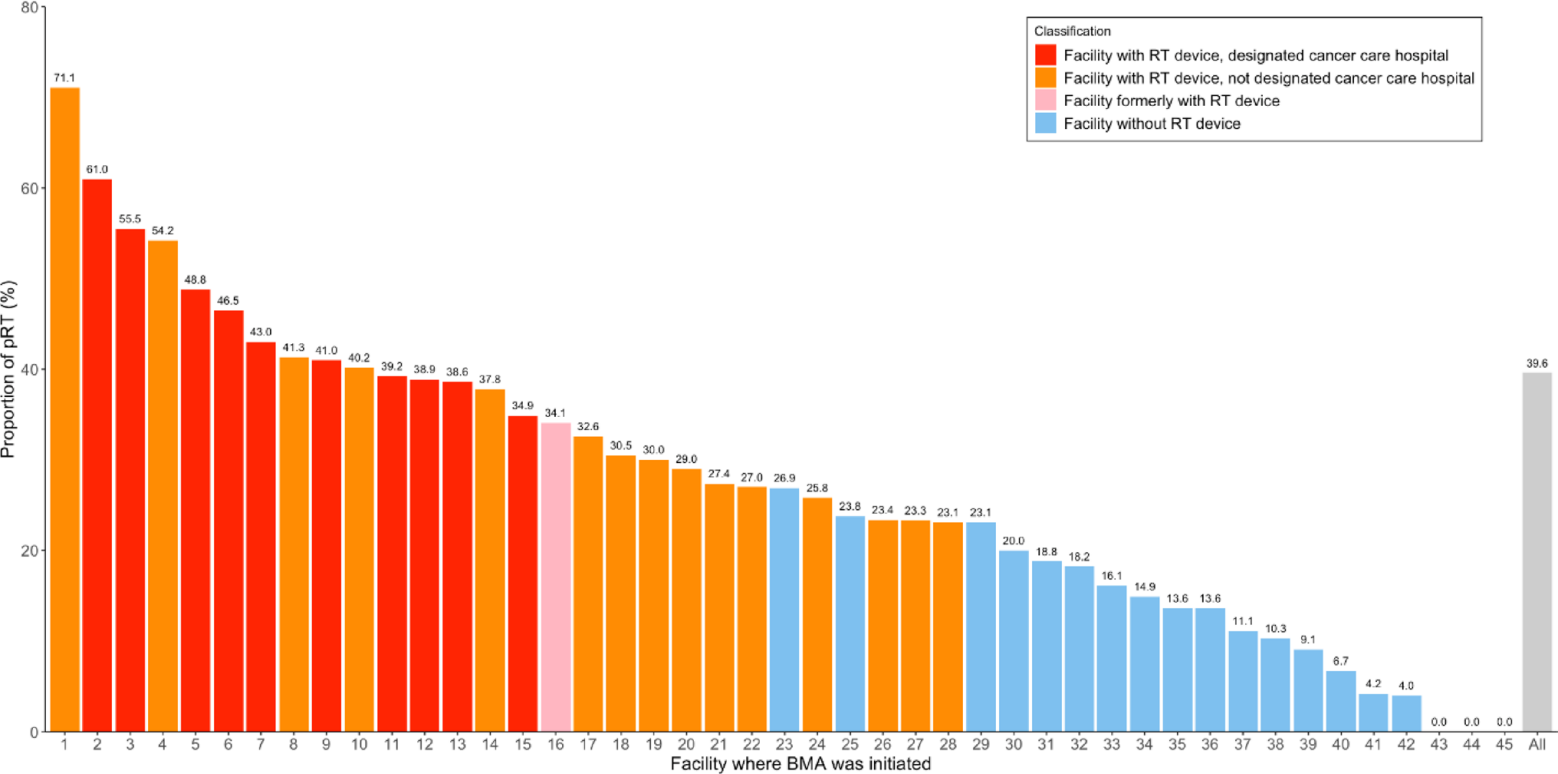
Proportion of patients who underwent pRT by the facilities where BMA was initiated. Numbers in the plotted areas represent individual data. pRT, palliative radiation therapy; BMA, body modifying agents

Furthermore, the proportion of patients treated with pRT was surveyed in facilities where BMA were initiated (Fig. 3). The proportions ranged from 0 to 71.1% for all BMA facilities, 23.1–71.1% for BMA facilities with RT devices, and 0–26.9% for BMA facilities without RT devices. The proportion of patients treated with pRT in the BMA facilities with and without RT device was 43.1% (95% CI 41.8–44.4%) and 11.7% (95% CI 9.5–14.3%), respectively. This difference was significant across the SMA (Table 3). After adjusting for age categories, malignancies, and SMA, the proportion of pRT in the facilities with RT devices was still significantly higher than those without RT device, with an odds ratio of 5.55 (95% CI 4.35–7.14). Patients treated with BMA in designated cancer care hospitals were likely to receive pRT than those in the other hospitals (47.7% [95% CI 46.2–49.3%] vs 26.7% [95% CI 24.9–28.5%], *p*-value < 0.001).

**Table 3:** Proportion of patients who underwent pRT by RT availability at the facilities for BMA

Place of residence, SMA	RT availability in facilities where BMA started	n	Percentage of pRT (95% CI)	RD (95% CI)	RR (95% CI)	*p*-value*
Seibu	Yes	1188	42.6 (39.8–45.4)	31.1 (24.3–37.8)	3.69 (2.16–6.31)	< 0.001
	No	104	11.5 (6.7–19.0)			
Chutoen	Yes	700	47.0 (43.3–50.7)	34.8 (27.3–42.2)	3.84 (2.25–6.56)	< 0.001
	No	98	12.2 (7.1–20.1)			
Shida-Haibara	Yes	651	36.1 (32.5–39.9)	22.1 (11.2–33.1)	2.59 (1.22–5.47)	0.003
	No	43	14.0 (6.6–27.3)			
Shizuoka	Yes	1123	36.0 (33.2–38.9)	24.7 (18.0–31.3)	3.18 (1.85–5.44)	< 0.001
	No	106	11.3 (6.6–18.7)			
Fuji	Yes	571	45.0 (41.0–49.1)	31.7 (20.9–42.4)	3.38 (1.59–7.15)	< 0.001
	No	45	13.3 (6.2–26.1)			
Sunto-Tagata	Yes	1003	49.5 (46.4–52.6)	37.3 (31.0–43.7)	4.08 (2.57–6.49)	< 0.001
	No	132	12.1 (7.6–18.8)			
Atami-Ito	Yes	208	54.8 (48.0–61.4)	45.0 (36.5–53.4)	5.57 (3.27–9.46)	< 0.001
	No	132	9.85 (5.8–16.1)			
Kamo	Yes	143	46.9 (38.9–55.1)	35.0 (22.2–47.7)	3.94 (1.70–9.13)	< 0.001
	No	42	11.9 (5.2–25.0)			
Overall	Yes	5587	43.1 (41.8–44.4)	31.4 (28.7–34.1)	3.69 (3.00–4.53)	< 0.001
	No	702	11.7 (9.5–14.3)			

pRT, palliative radiation therapy; BMA, bone-modifying agent; SMA, secondary medical area; RD, risk difference; CI, confidence interval; RR, risk ratio

*Fisher’s exact test

## Discussion

To the best of our knowledge, this is the first study to evaluate practice disparities in pRT for bone metastases in a Japanese population-based setting. In the SKDB cohort, 39.6% of BMA-treated patients with bone metastases were treated with pRT. The proportion of pRT differs among SMA with a range of 33.8–45.1%, whereas a wider range among BMA facilities, 0–71.1% among all facilities, 23.1–71.1% among BMA facilities with RT devices, and 0–26.9% among BMA facilities without RT device. In BMA facilities with and without RT device, pRT was delivered to 43.1% and 11.7% of patients, respectively. Patients treated with BMA in the designated cancer care hospitals were more likely to receive pRT.

In this study, patients who had potential indications of pRT for bone metastases was defined as patients with an ICD-10 code of C79.5 “Secondary malignant neoplasm of bone and bone marrow” and had initiated BMA including zoledronic acid or denosumab. While no validation study is available for this definition, it is considered sufficiently specific for identifying bone metastases, as it is based on the prescription of the BMA which are indicated only for malignant tumors in Japan. However, it should be noted that our cohort included asymptomatic bone metastases some of which were not indicative of pRT. Conversely, patients with bone metastases who were not treated with BMA, and/or who lacked the code of C79.5 were not included. These factors contribute to the discrepancy between our definition of potential indication of pRT and the actual disease status. Nevertheless, we consider that any influence on the main findings of our study is minimal and does not affect the conclusions regarding regional and institutional disparities in pRT for bone metastases.

Additionally, we should discuss the claim-based definition of pRT. The purpose of RT is almost unidentified in Japanese administrative claims codes themselves. Therefore, we limited the analysis period to 3 months following BMA administration to identify RT that could be considered pRT for bone metastases. Even when using this definition, there remained a possibility that RT to other sites could be recognized as pRT for bone metastases. However, supplemental analysis showed that 79.9% (2,490 out of 3,140) of RT was performed within 3 months following BMA initiation, which means our definition of “pRT for bone metastases” has reasonable validity.

We examined in detail whether the proportion of patients receiving pRT differed according to SMA. However, despite some areas had no facilities with RT devices, no large differences were observed among the SMA. High-volume facilities, such as a cancer center or university hospitals in adjacent areas, may have provided the demand for pRT in such areas. This hypothesis was supported by our finding that the proportion of pRT deliveries was higher in designated cancer care hospitals.

Among the factors related to the delivery of pRT, across all SMA, the on-site availability of RT at facilities where BMA were initiated was consistently associated with a higher proportion of pRT delivery compared to facilities lacking RT devices, which is consistent with those of a previous study that used data from the Ontario Cancer Registry [7]. Regardless of differences in healthcare systems, geography, or other factors, the lack of RT devices in hospitals primarily responsible for a patient’s cancer treatment is a challenge to the use of pRT.

Even when the proportion of pRT delivery was evaluated separately for BMA facilities with and without RT devices, the differences between facilities were still significant. Especially, the higher proportions of pRT deliveries were observed in designated cancer care hospitals. As such kind of hospitals are required to employ full-time radiation oncologists, to hold tumor bord, and to have medical care collaboration networks, they could improve the delivery of pRT.

## Limitation

This study had several limitations. First, this study was conducted only among residents of Shizuoka Prefecture, Japan; therefore, it is unknown whether our results can be extrapolated to other countries or regions.

Second, as we defined the potential indication for pRT as patients who had an ICD-10 code of C79.5″ Secondary malignant neoplasm of bone and bone marrow” and were started on zoledronic acid or denosumab, there were patients who did not meet our definition but were eligible for pRT, as mentioned above. Given the inherent limitations of claims data, it is not feasible to identify a population indicative of pRT for bone metastases with complete accuracy. To reduce misclassification, we employed strict inclusion and exclusion criteria designed to enhance the specificity of case identification. While this approach may have resulted in the exclusion of some patients who needed pRT for bone metastases, we consider it unlikely that the treatment patterns among excluded cases differ substantially from those included. Thus, we judged that reducing false-positive classification was more important than maximizing the study population.

Third, the definition of pRT in this study was based on medical claims data and may not fully reflect actual clinical administration. However, considering that most RT was initiated within the same month of BMA initiation (Fig. S2), it is reasonable to interpret most of these RTs as being intended for bone metastases. This interpretation is further supported by previous population-based studies reporting that patients with bone metastases may concurrently have brain or lymph node metastases, with prevalences of approximately 10–20% in breast [14], lung [15, 16], and kidney [17] cancers, and ≤ 5% in prostate cancer [18]. These findings suggest that some degree of misclassification is unavoidable when using administrative claims data. Nevertheless, because the proportion of such cases is relatively small, substantial misclassification is unlikely to have influenced our overall findings. Fractionation data of RT would be helpful in identifying the aim of RT; however, we were unable to use these data because the number of RT sessions recorded in the SKDB may be duplicated in some inpatient cases due to administrative processing under the DPC system. As no validated correction method currently exists, we did not include fractionation patterns in this study. Additionally, pRT prior to the month wherein BMA was initiated was not evaluated, but this may not matter when evaluating disparities among areas or facilities.

Fourth, it may be difficult to account for differences in pRT according to malignancy, as malignant neoplasms were identified using ICD-10 codes during the baseline period and may not be indicative of a primary tumor. In patients with multiple malignancies, the primary tumor could not be identified in the present study. In addition, the indications for BMA in clinical practice may differ according to the primary tumor, which may affect our definition of the potential indications for pRT.

Fifth, the SKDB does not have information on patients’ socioeconomic status, which is reportedly a factor associated with the use of pRT [16], but the socioeconomic impact may not be as great in Japan, which has a universal health insurance system.

Sixth, the SKDB has insufficient data on patient status, such as performance status, clinical stage, and comorbidities, which might influence pRT delivery. Finally, our definition of pRT did not include IMRT, SBRT, or particle therapy, excluding RT, which is not primarily aimed at palliation. However, as the usefulness of high-precision RT for bone metastases has been recently reported [19], studies on high-precision pRT are required.

Despite these limitations, the present study provides information on the large disparities among facilities in the delivery of pRT for bone metastases, indicating the need for attempting to equalize the clinical practice of pRT.

## Conclusion

In the regional population-based setting, the proportion of patients with bone metastasis who underwent pRT was not greatly different among the SMA, but significantly different among the facilities where the BMA was initiated. This study contributes to providing an equal opportunity to receive pRT regardless of the patients’ residence and facilities where they are treated for bone metastases.

## Supplementary Information

Below is the link to the electronic supplementary material.

Supplementary Material 1

## Abbreviations

BMA: Bone-modifying agents
CI: Confidence interval
ICD-10: International Classification of Disease, tenth edition
IMRT: Intensity-modulated radiation therapy
MHLW: The Ministry of Health, Labour, and Welfare
pRT: Palliative radiation therapy
RD: Risk difference
RR: Risk ratio
RT: Radiation therapy
SBRT: Stereotactic body radiation therapy
SMA: Secondary medical areas
SREs: Skeletal-related events
