# Supplementary material for: Practice disparities in palliative radiation therapy for bone metastases: insights from the Shizuoka Kokuho database study
Source: Jpn J Radiol. 2026 Jan 14;44(6):1071–8. doi: 10.1007/s11604-025-01938-8 (PMC13222301; doi:10.1007/s11604-025-01938-8)
Supplement: Supplementary file 1 — Supplementary Material 1 [file 11604_2025_1938_MOESM1_ESM.pdf]

Supplementary Information

Practice Disparities in Palliative Radiation Therapy for Bone Metastases: Insights from the Shizuoka Kokuho Database Study

Japanese Journal of Radiology

Yuhei Miyasaka, Yoko Sato, Hideyuki Harada, Katsumasa Nakamura, Tatsuya Ohno, Seiichiro Yamamoto

Corresponding author: Seiichiro Yamamoto

Graduate School of Public Health, Shizuoka Graduate University of Public Health

E-mail: syamamoto@s-sph.ac.jp

Supplementary Table 1 (Table S1): List of claims codes for BMA

BMA	Code
Denosumab	622136501
Zoledronic acid	622342601, 622342603, 622342701, 622342703, 621657601, 622216901, 622337201, 622337301, 622338001, 622344201, 622344301, 622351301, 622351402, 622354601, 622354701, 622355401, 622356301, 622358301, 622358401, 622360301, 622360401, 622391001, 622388201, 622351401, 622342701, 622621900, 622385901

Abbreviation: BMA, bone-modifying agents

Supplementary Table 2 (Table S2): List of ICD-10 codes for baseline characteristics

Malignancies	ICD-10 code
Head and neck	C00–C09, C11–
Esophagus	C15
Stomach	C16
Colorectum	C18–C20
Kidney	C64, C65
Liver	C22–C24
Pancreas	C25
Lung	C33, C34
Skin	C43, C44
Breast	C50
Uterine cervix	C53
Endometrium	C54
Ovarian	C56
Prostate	C61
Bladder	C67
Thyroid	C73
Malignant lymphoma	C81–C86
Multiple myeloma	C90

Abbreviation: ICD-10, International Classification of Disease, Tenth edition

Supplementary Table 3 (Table S3): List of procedure code for RT

Section code	Procedure	Procedure code
M001-00	EBRT (high-energy), 1 field	180020710
	EBRT (high-energy), 2 fields, opposing	180020810
	EBRT (high-energy), 2 fields, not opposing	180020910
	EBRT (high-energy), 3 fields	180021010
	EBRT (high-energy), 4 fields or more	180021110
	EBRT (high-energy), moving field	180021210
	EBRT (high-energy), conformal	180021310
	EBRT (high-energy), 1 field *	180021410
	EBRT (high-energy), 2 fields, opposing*	180021510
	EBRT (high-energy), 2 fields, not opposing*	180021610
	EBRT (high-energy), 3 fields*	180021710
	EBRT (high-energy), 4 fields or more*	180021810
	EBRT (high-energy), moving field*	180021910
	EBRT (high-energy), conformal*	180022010
M001-03	RT with a linear accelerator, not SBRT (a series of RT)	180035310

* Second session of the day

Abbreviations: RT, radiation therapy; EBRT, external beam radiation therapy; SBRT, stereotactic body radiation therapy

Supplementary Table 4 (Table S4): Numbers of patients by year

Year	Number of included patients	Number of patients who underwent pRT	%
2012	128	32	25.0
2013	587	184	31.3
2014	661	250	37.8
2015	689	286	41.5
2016	661	271	41.0
2017	639	274	42.9
2018	622	260	41.8
2019	650	267	41.1
2020	643	261	40.6
2021	601	235	39.1
2022	408	170	41.7

Abbreviation: pRT, palliative radiation therapy

Supplementary Figure 1 (Fig. S1): Flow diagram of the included patients

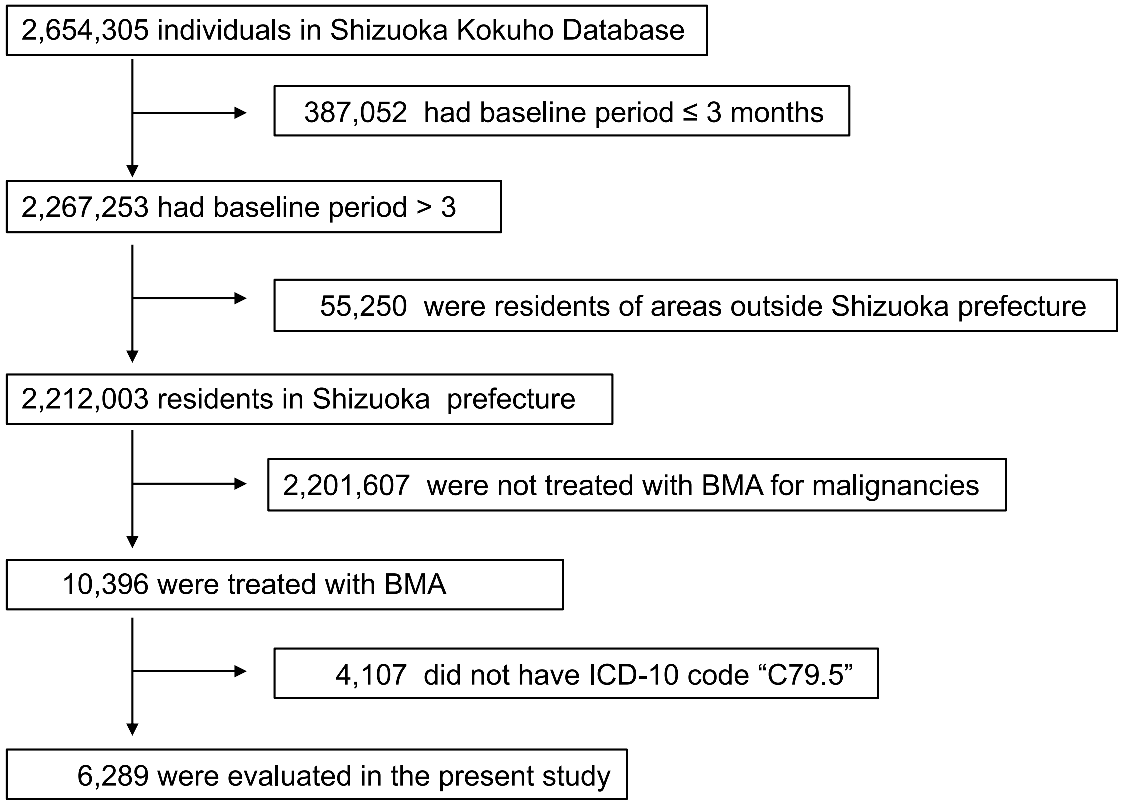

Abbreviations: BMA, bone-modifying agent; ICD-10, International Classification of Disease, Tenth edition

Supplementary Figure 2 (Fig. S2): Numbers of RT after the initiation of BMA per month

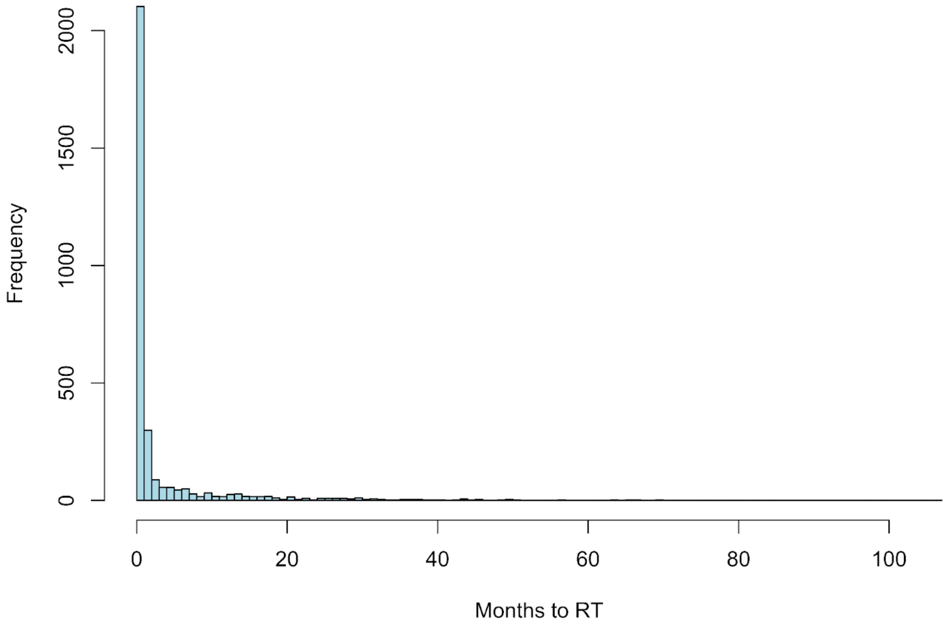

Abbreviations: RT, radiation therapy; BMA, bone-modifying agents
